# Shade avoidance responses in seedlings of maize hybrids representing 100 years of breeding history

**DOI:** 10.3389/fpls.2026.1939000

**Published:** 2026-09-30

**Authors:** Memiş Bilgici, Sara Lira, Lucas Borras, Elnaz Ebrahimi, Kenneth J. Moore, D. Raj Raman, Thomas Lübberstedt

**Affiliations:** 1 Department of Agronomy, Iowa State University, Ames, IA, United States; 2 Corteva Agriscience, Johnston, IA, United States; 3 Department of Agricultural and Biosystems Engineering, Iowa State University, Ames, IA, United States

**Keywords:** far-red light, cover crops, intercropping, hybrid, maize, shade avoidance, stomata, long-term breeding

## Abstract

Understanding how the spectral quality of light affects maize physiology is critical, as it mediates an early response to competition. We measured the effects of light quality on physiological traits at the seedling stage in 27 maize genotypes released from 1920 to 2022. High and low red/far-red ratios light conditions were simulated in a growth chamber. Low R/FR ratio caused shade avoidance responses, including increased plant height and decreased stem diameter. We hypothesized that long term selection for yield would reduce phenotypic response to low R/FR light conditions in seedling stages because more modern hybrids have been selected under progressively higher planting densities. While changes in phenotype, including reduced leaf area, root length, total stomatal pore area and increased leaf SPAD index, were observed over past 100 years breeding time, for most traits, no significant genotype x light treatment interaction (p > 0.05; F = 0.64-1.00) for any trait. Selection has likely improved resource use efficiency for other factors affected by plant-to-plant competition, such as water or nitrogen.

## Highlights

- To our knowledge, this is the first study to examine how shade avoidance responses have changed over the past 100 years of maize breeding. Low red/far-red ratio light induced shade avoidance responses in maize seedlings. Seedling plant height increased, while stem diameter decreased under low R/FR conditions. Maize hybrids released from 1920 to 2022 differed in key physiological traits. Long term-breeding of maize affected leaf area, root length, SPAD index, and stomatal traits.

## 1 Introduction

Maize (*Zea mays L*.) is an important global crop, essential for food, fuel, and animal feed production. Over the past 100 years, U.S. maize grain yields have increased more than 7-fold due to the combined effect of long-term breeding and agronomic management changes (Duvick, 2005a; Rizzo et al., 2022; Ruiz et al., 2026). However, maize production as a monoculture or in limited crop rotations has led to soil degradation, water pollution, and biodiversity loss (Karlen et al., 2013; Cárceles Rodríguez et al., 2022).

Intercropping with perennial ground covers (PGC) between maize rows can help to mitigate these issues by reducing soil erosion, improving soil health and promoting biodiversity (Moore et al., 2019). Production efficiency can be improved by partnering maize with ecologically complementary perennial species growing during different seasons, root in different parts of the soil profile, or using nutrients at different times (Seran and Brintha, 2010; Chen et al., 2022). Managing resource competition between maize and PGC remains a challenge (Schlautman et al., 2021; Bartel et al., 2022; Moore et al., 2022), particularly during early maize growing stages. During this period maize seedlings compete with cover crops for light, nutrients and water (**Supplementary Figures 1**, **2**). Water stress may be alleviated by irrigation, and light competition can be reduced through timely suppression and maintenance of an adequate crop growth zone. However, in poorly-suppressed or unsuppressed systems, competition for light, particularly in terms of light quality, is unavoidable (Rajcan et al., 2004; Tokatlidis, 2017).

The ratio of red (R) to far-red (FR) light reflected from the soil and neighboring plants significantly impacts seedling growth (Kasperbauer and Karlen, 1994; Wang et al., 2016). Direct sunlight has a high R/FR ratio, while reflectance and absorption by leaves cause this ratio to decrease deeper within the canopy (Jafari et al., 2024; Zhou et al., 2024). Plants have evolved two fundamentally different strategies to cope with low R/FR light: shade avoidance response (SAR) and shade tolerance (ST) (Maddonni et al., 2002; Page et al., 2010). In maize, SAR, also called shade avoidance syndrome (SAS), occurs in maize during early vegetative growth in response to reduced (R/FR) light ratios. During early developmental stages, SAR leads to rapid stem elongation, redirection of resources from roots to shoots, changes in leaf angle or orientation. At later stages, SAR leads to early flowering, and accelerated senescence, all aimed to outcompete neighboring plants (Hu et al., 2023; Yan et al., 2024). SAR is triggered by spectral shifts due to reflection and absorption by other species (e.g., PGC, or weeds, or an annual cover crop) as well as by other maize plants, such as in high-density plantings (Collison et al., 2020; Menalled et al., 2024).

In contrast to SAR, ST represents a physiological acclimation to a growth environment rather than an escape. Plants selected for ST have more compact architecture, invest in efficient light capture and utilization, sustain balanced root-shoot ratios, and preserve photosynthetic capacity under reduced R/FR regimes (Gommers et al., 2013; Xu et al., 2021; Casal and Fankhauser, 2023). Maize genotypes with ST show minimal SAR, accumulate a higher proportion of ear biomass during flowering despite competition, maintain stable growth rates under stress (Borrás and Vitantonio-Mazzini, 2018) and succeed by functioning effectively within shade, not by avoiding it.

Modern high-yielding maize hybrids thrive at much higher planting densities than older varieties (Mansfield and Mumm, 2014; York et al., 2015; Shi et al., 2024) due to upright leaf angles, smaller tassels, decreased plant-to-plant variability, and better biomass partitioning to ears during flowering (Tollenaar and Wu, 1999; Campos et al., 2004; Messina et al., 2022; Tian et al., 2024; Elli et al., 2025; Reyes et al., 2026). As a consequence, maize planting densities have increased from 25,000 to 90,000 plants ha^−1^, a 3.6-fold over the last century (Lee and Tollenaar, 2007; USDA-NASS, 2025). Crowding tolerance has been a key driver for increasing the crop productivity (Borras et al., 2026). A critical temporal difference distinguishes the progression of R/FR received by maize in conventional monoculture from that received when a cover crop has not been suppressed. In conventional maize fields, seedlings emerge in bare soil with high R/FR ratios. R/FR rates decrease gradually as the canopy closes, giving plants time to acclimate. Well managed PGC systems have a relatively short and well-suppressed groundcover, thereby minimizing the risk of triggering SAR. However, if suppression fails or is delayed or the crop is planted with a green cover crop, maize seedlings are exposed to low R/FR, and maize with low SAR may help to mitigate any negative response and yield losses.

Light quality responses involve multiple physiological systems. Beyond morphological changes, stomatal responses to light quality add another layer of complexity. Stomata are essential for photosynthesis as they enable the exchange of gases between leaves and the atmosphere (Lawson and Blatt, 2014). Stomata are usually open under well-watered conditions, allowing uptake of carbon dioxide (CO_2_) and release of water (H_2_O) and oxygen (O_2_). Stomata continuously adjust their opening to maintain a balance between carbon gain and water conservation, rather than being simply fully open or closed (Matthews et al., 2020; Lawson and Matthews, 2020; Serna, 2022; Chen et al., 2024). Moreover, the density of stomata on leaves as well as stomata size can be affected by external factors, such as light quality (Tan et al., 2022; Mukhtar et al., 2026).

Our central question was whether a century of long-term breeding for higher yield and increased planting densities inadvertently altered maize seedling response to shade signals. We used a set of genotypes from the Pioneer ERA genotypes collection representing 100 years of long-term breeding for yield (from 1920s to 2020s) plus representative current commercial hybrids, to study changes in shade responses over time. Our objectives were (i) to quantify maize seedling trait responses to both high (control) and low R/FR (shade) ratios, for key phenotypic, morphological, and stomatal traits, (ii) to quantify trends of seedling traits by estimating the association between hybrid release year and trait values under high R/FR (control) and under low R/FR (shade) separately, and (iii) to test whether the magnitude and form of the shade response has changed with release year.

## 2 Materials and methods

### 2.1 Plant materials

We used 27 genotypes (hybrids) with release years spanning 1920 to 2022. Twenty-six were commercial hybrids from the Pioneer ERA collection. The remaining entry, Reid Yellow Dent (1920), is an open-pollinated cultivar grown in the central U.S. Midwest before commercial hybrids became available, included here as a pre-hybrid reference point. For era comparisons, genotypes were classified as “historic” (n = 14, released 1920-2012) or “modern” (n = 13, released 2013-2022). The historic group therefore spans 92 years and the modern group 10 years; this imbalance is addressed in the sensitivity analyses; to compare breeding eras, the analysis was repeated with breeding period fitted as a categorical fixed effect, using the classification historic (1920-2012) and modern (2013-2022). Because Reid Yellow Dent is neither a hybrid nor part of the ERA series, all release-year regressions and era comparisons were additionally run with this entry excluded (Bilgici et al., 2026).

### 2.2 Growth chamber experiments

Experiments were conducted in two identical growth chambers (CONVIRON Flex; 3.6 m^2^ growth area, 635 mm growth height) equipped with LED arrays rated to deliver up to 1000 µmol m^−2^ s^−1^ at 152 mm below the fixture; low R/FR (shade) ratio and regular high R/FR ratio light (control). Fixtures were dimmed so that both chambers delivered a PPFD (400–700 nm) of 743 µmol m^−2^ s^−1^ at canopy height, verified with a quantum light meter (Apogee Instruments, Logan, UT, USA). The low R/FR treatment had an R/FR ratio of 0.61 (182 µmol m^−2^ s^−1^ red, 600–700 nm/300 µmol m^−2^ s^−1^ far-red, 700–800 nm) and the control had a ratio of 2.04 (323/158 µmol m^−2^ s^−1^). The control ratio is higher than the value typical of open-field sunlight at midday (R/FR > 1.2) because the LED array was configured to maximize the R/FR contrast while holding PPFD constant. PPFD was identical in the two chambers, this is a pure light-quality contrast. This study compared responses to a high R/FR ratio (control) vs low R/FR ratio (shade) contrast rather than strictly open field sunlight vs shade. Thus, two well-defined R/FR light environments (0.61 and 2.04) and differing only in the red-far-red photon ratio, were applied to the 27 maize genotypes.

Temperatures were maintained at 25°/15 °C (day/night), with a light cycle of 14/10 h (day/night). Relative humidity was 65% ± 5%. The chambers were regularly monitored to verify that proper temperature, humidity, and light intensity were maintained. Planting trays (15-cell trays, 127 mm deep) were filled with moistened Berger BM7 bark mix soil containing a starter fertilizer, bark, coarse perlite, dolomitic and calcitic limestone, premium coarse peat moss, and a non-ionic wetting agent. Maize seeds were planted at a depth of approximately 20 mm. One seed per cell was sown in 15-cell (3 × 5) trays (32.7 cm × 53.8 cm; cell depth ~12 cm), resulting in a planting density of 85 plants m^−2^ (15 plants per tray) with approximate within-tray spacing of 10.9 cm × 10.8 cm. Tap water was applied as needed every other day to keep the soil moist without waterlogging.

The experiment was repeated as three independent temporal runs. Within each run, one chamber was set to low R/FR and the other to high R/FR, giving a 3 runs × 2 light conditions structure. Because each light condition was housed in the same chamber throughout, the three runs are temporal replicates of the treatment rather than independent chamber-level replicates; this constraint is addressed explicitly in the Discussion. Within each chamber and run, a randomized complete block design (RCBD) was used, with the chamber layout divided into three blocks to account for positional gradients, resulting in three replications per run. Within each block, all 27 genotypes were represented once, and entry positions were randomized. Randomization was performed independently for each experiment. The experimental unit was a “plot” consisting of three adjacent plants. Thus, for each of the 27 genotypes within each experiment, there were three plots, one per block, totaling nine plants per genotype per experiment. When measurements were taken at the individual-plant level, values from the three plants within a plot were averaged to a mean per plot value prior to statistical analysis.

### 2.3 Plant measurements

Plant measurements were taken 14 days after planting across all genotypes, when the seedlings reached the four to five leaf stages (V4-V5) (Bilgici et al., 2026). Plant height (from soil level to the tallest point), root length (cm) (the roots were carefully removed from the tray-cells and cleaned of adhering growth medium. Root length was measured from the crown/base of the seedling to the tip of the longest root after gently extending the root system without stretching the roots therefore representing maximum root length). Cells were 127 mm deep, so the longest roots reached the base of the cell and continued to elongate along the cell wall; root length is therefore a measure of total elongation of the longest root. It does not quantify lateral root proliferation, root biomass, root surface area, branching, or mature nodal root architecture and should therefore be interpreted specifically as an early seedling root-extension phenotype. Stem (stalk) diameter (recorded at specific cross-sectional points using digital Vernier caliper, and averaged for accuracy) leaf stage, and presence of anthocyanin pigmentation on stems (marked as present or absent) were recorded. Leaf area (LA) of the second leaf was estimated using a ruler by measuring leaf length (L; from the ligule/collar to the leaf tip along the midrib) and leaf width (W; measured perpendicular to the midrib at the widest point of the lamina, excluding the sheath). Leaf area was calculated as Plant leaf length∗leaf width∗0.75 (Elings, 2000). Relative leaf chlorophyll content was measured on second leaf, midway along the blade and midway between the midrib and the margin with a SPAD meter (%) (SPAD-502Plus; 2021, Konica Minolta, Inc., Ramsey, NJ, USA); three readings per plant were averaged. They are therefore reported throughout as the SPAD index. For biomass measurements, shoots were collected after 14 days at the V4-V5 growth stage and oven-dried at 70 °C until constant weight (~72 h) before weighing. The dry biomass was weighed using a precision balance.

### 2.4 Stomatal measurements

For stomatal measurements on the second leaf for all genotypes (for details see Bilgici et al., 2026), a thin layer of clear nail polish was applied to the abaxial (lower) epidermis at the widest point of the blade and allowed to dry completely. A strip of Scotch^®^ Transparent Tape (3M, MN, USA) was pressed onto the dried area and peeled away, lifting a cast of the epidermis containing stomatal imprints (Bilgici et al., 2026). The tape was mounted on a glass slide for microscopic observations.

Microscopy was performed using an Olympus BX40 microscope equipped with a high-definition camera, trinocular viewing head, C-mount adapter, and 40 x/0.65 Ph_2_ ¥/0.17 objective lens and 10x ocular lens oil-immersion objective.

### 2.5 Data analysis

All statistical analyses were conducted using R software 4.4.3 (R Core Team, 2024) with RStudio (R Core Team, 2024) (http://www.r-project.org). Calculations were performed using the lmer package in the *lme*4 R package (Bates et al., 2015) and *nlme* (Pinheiro et al., 2017). Figures were created for data visualization using the package ggplot2 (Wickham, 2016). To evaluate temporal trends in maize phenotypes across the breeding period from 1920 to 2022 and to test the effect of light environment, the following model was fitted for each trait:


$${\text{Y}}_{\text{ijkb}}=\mu +\beta_{1}\;{\text{Year}}_{\text{i}}+{\text{L}}_{\text{i}}+\beta_{2}\left({\text{Year}}_{\text{i}}\times {\text{L}}_{\text{i}}\right)+{\text{G}}_{\text{i}}+{\text{R}}_{\text{k}}+{\text{C}}_{\text{b}\left(\text{jk}\right)}+\epsilon_{\text{ijkb}}$$

where: *Tijkb* is the observed phenotype of genotype *i* under light treatment *j*, in experiment run *k*, replicate *b*; *u* is the overall mean; *Yeari* is the release year of genotype *i*, fitted as a continuous fixed effect; *Lj* is the fixed effect of light treatment (high or low R/FR); *Yeari x Lj* is fixed interaction testing whether the release-year trend differs between light conditions (high and low R/FR). *Gi* is the random effect of genotype, *Rk* is the random effect of run (*k* = 1,2,3), *Cb*(*jk*) is the random effect of replicate/block nested within run and light (*b* = 1,2,3) represented the chamber-level experimental unit for the light treatment, *eijkb* is the residual. For tests of genotype’s main effect, we used the Genotype x Run interaction as the error term (denominator), which evaluates whether genotypes’ differences are consistent-stable across replicated runs. The same model structure was used for all traits. To compare breeding eras, the analysis was additionally repeated with breeding period fitted as a categorical fixed effect, where genotypes released from 1920 to 2012 were classified as historic and those released from 2013 to 2022 as modern hybrids. Mixed model analysis, the t-test (student) and Tukey’s test were used for significance tests between low and high R/FR ratio light treatments. Pearson correlation coefficients were calculated to analyze relationships between the evaluated traits. Relationships between the year of release and traits were analyzed using linear least-squares regression. R^2^ calculations of marginal and conditional effects for the models were performed using the “performance” package (Lüdecke et al., 2021). For directional response we computed, per genotype, the PCA displacement vector (low and high R/FR ratio) and tested its mean against zero with one-sample Hotelling’s *T*^2^. Displacement magnitude (“PCA shift length”) was defined as


$$\sqrt{{\left(\Delta \text{PC}1\right)}^{2}+{\left(\Delta \text{PC}2\right)}^{2}}$$

and regressed on year of release. Rank associations were assessed by Spearman’s ρ. Historic genotypes and modern hybrids were compared by Welch’s *t*-test and Wilcoxon rank-sum implemented in the package’s vegan (Oksanen, 2015) and with mixed-model hypothesis tests in lmerTest (Kuznetsova et al., 2017) to test whether modern hybrids differ from older genotypes in plasticity,


(Δ Trait=low R/FR−high R/FR)

and whether release year × light condition interactions were significant. Using lmer package in the *lme*4 R package, linear random-effects models were employed to determine Best Linear Unbiased Predictors (BLUPs) for each genotype’s maize traits. Genotype was fitted as a fixed effect for hypothesis testing and as a random effect in a separate model to obtain BLUPs and heritability estimates. Genotype BLUPs for all traits under both light conditions are reported in **Supplementary Table 1**. The model was fitted using the restricted maximum likelihood method. For each light environment in **Table 1**, Broad-sense heritability (H^2^) on an entry-mean basis was calculated as:

**Table 1:** Response of plant architecture and composition traits to high R/FR (control) and low (shade) ratios across 27 maize genotypes with broad-sense heritability estimates (H^2^) and 95% confidence intervals (CI).

	Mean			F-value			Broad sense heritability (H^2^)	
Trait	Df	Low R/FR ratio	High R/FR ratio	Light condition	Genotype	Genotype x light condition	High R/FR with CI lower-upper	Low R/FR with CI lower-upper
Plant height (cm)	26	30.94	26.46	86.07***	3.14***	0.74 n.s.	0.76 (0.63-0.82)	0.57 (0.38-0.69)
Leaf area (cm^2^)	26	20.41	19.25	27.35***	38.85***	1.00 n.s.	0.96 (0.91-0.98)	0.86 (0.77-0.89)
Stem (stalk) diameter (mm)	26	0.47	0.50	19.16***	1.93**	0.77 n.s.	0.76 (0.64-0.82)	0.54 (0.41-0.63)
Root length (cm)	26	19.88	19.37	6.82**	8.48***	0.89 n.s.	0.88 (0.82-0.91)	0.86 (0.77-0.89)
SPAD index	26	36.20	35.06	7.52**	3.09***	0.64 n.s.	0.66 (0.46-0.75)	0.62 (0.43-0.73)
Dry biomass (g)	26	0.29	0.28	0.61 n.s.	3.69***	0.92 n.s.	0.75 (0.62-0.82)	0.66 (0.46-0.76)

**p< 0.01, ***p< 0.001; n.s., not significant. 95% Confidence Interval (CI).


$$H^{2}=\sigma_{g}^{2}/\left(\sigma_{g}^{2}+\frac{\sigma^{2}GE}{E}+\frac{\sigma^{2}E}{rE}\right),$$

where $\sigma_{g}^{2}$ represents the genotypic variance, σ^2^ the error variance, σ^2^*GE* the genotype-by-environment interaction variance, *E* the number of lights (*E* = 2), σ^2^*E* the residual (error) variance and *r* the number of replications per light environment (r = 9; three blocks in each of three runs in the growth chambers per experiment.

## 3 Results

### 3.1 Shade avoidance response of seedling plant architecture traits

Most maize seedling traits were significantly influenced by both genotype and R/FR ratio, while no significant genotype x light environment interactions were found for any of the traits (**Table 1**). Genotypes were significant for all traits, and the light treatment impacted plant height, leaf area, SPAD indexes, root length, and stem (stalk) diameter, but not dry biomass. Under low R/FR conditions, plant height, leaf area, root length, and SPAD indexes increased, while stem (stalk) diameter decreased. Long-term trends for the year of hybrid release showed consistent decreases in leaf area, root length, and root-to-plant height ratio, while SPAD indexes increased significantly from 1920 to 2022 (**Figure 1**).

**Figure 1:**
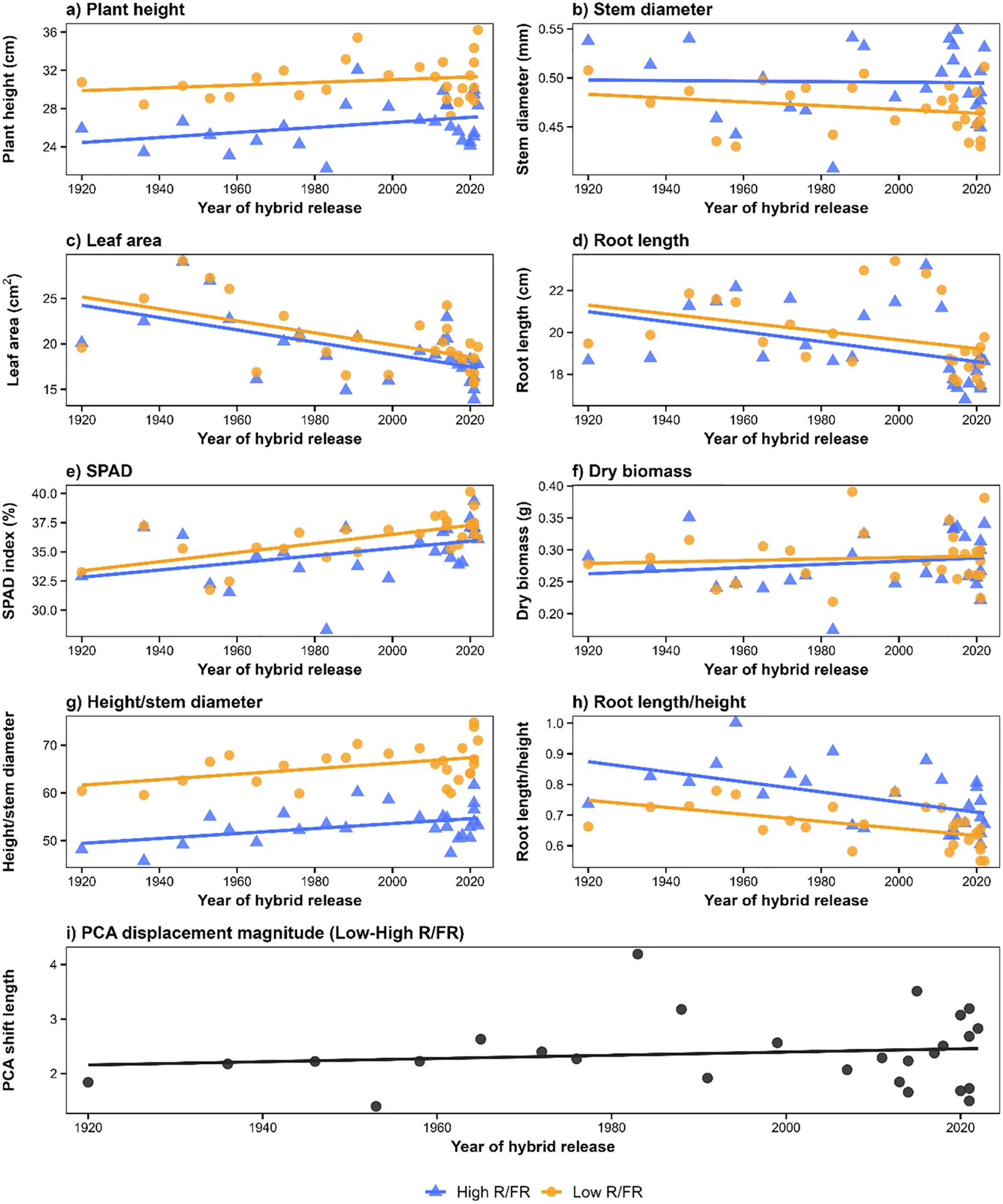
Trends in seedling traits and shade-response magnitude across 27 maize genotypes with different year of release grown under high and low R/FR ratios light conditions. **(a–h)** Relationships between year of release and mean seedling traits measured under high (control) R/FR (blue) and low (shade) R/FR (orange): **(a)** plant height, **(b)** stem (stalk) diameter, **(c)** leaf area, **(d)** root length, **(e)** SPAD index, **(f)** dry biomass, **(g)** plant height/stem diameter ratio, and **(h)** root length/plant height ratio. Points are genotype means and lines are linear regressions fitted separately for each light treatment. Across traits, slopes under high and low R/FR are largely parallel, indicating that long-term breeding changed trait means similarly in both light R/FR ratios environments rather than specifically under shade. (Bottom panel) **(i)** Shade response magnitude. The multivariate shade response for each genotype was summarized as the Euclidean distance (“PCA shift length”) between its positions under high (control) and low (shade) R/FR ratios in PCA trait space.

At a low compared to high R/FR ratio, seedling plant height increased by 4.48 cm, stem (stalk) diameter decreased by 0.03 mm, leaf area increased by 1.16 cm^2^, root length increased by 0.51 cm, and SPAD index increased by 1.14 units (**Table 1**, **Supplementary Figures 3**-**5**). All of these changes were statistically significant (p< 0.01). Broad sense heritabilities (H^2^) for maize seedling traits ranged from 0.54 to 0.96. The highest heritability (0.96) with a narrow confidence interval (CI 0.91-0.98) was observed for leaf area under high R/FR. Conversely, the lowest heritability (0.54) (CI 0.41-0.63) was found for stem (stalk) diameter under low R/FR. For all traits, heritabilities were higher under high R/FR compared to low R/FR conditions (**Table 1**).

Under both high and low R/FR conditions, root length (cm) decreased in modern genotypes, with reductions of 2.51 cm (p< 0.001) for high R/FR and 2.09 cm (p< 0.001) for low R/FR (**Figure 1**). SPAD indexes significantly increased in modern genotypes under both conditions. The increase was 2.32 units (p< 0.01) for high R/FR and 1.80% (p< 0.05) for low R/FR. Leaf area decreased significantly under low R/FR conditions, with a reduction of 2.67 cm^2^ (p< 0.05). Under high R/FR conditions, the decrease was 2.6 cm^2^ (p< 0.05). Only at high R/FR, modern hybrids showed significantly increased dry biomass. Modern and historic genotypes did not differ for dry biomass, plant height and stem (stalk) diameter.

### 3.2 Correlations of plant architecture between low and high R/FR ratios

Traits measured under high and low R/FR ratios were positively correlated across environments for all major seedling traits. However, the strength of these relationships differed among traits (**Table 2**). The strongest cross-environment correlations were observed for leaf area (r = 0.96, p< 0.001) and root length (r = 0.88, p< 0.001), indicating very high consistency in genotype performance between light regimes. Significant positive correlations were also detected for SPAD (r = 0.72, p< 0.001), plant height (r = 0.62, p< 0.001), stem (stalk) diameter (r = 0.59, p< 0.01), and dry biomass (r = 0.59, p< 0.01). In contrast, most cross-trait correlations between environments were weak or non-significant. A few notable exceptions were observed: plant height under high R/FR was positively associated with stem diameter (r = 0.39, p< 0.05) and dry biomass (r = 0.53, p< 0.01) under low R/FR; stem (stalk) diameter under High R/FR was positively associated with dry biomass under low R/FR (r = 0.63, p< 0.001); and root length under high R/FR was positively associated with leaf area under low R/FR (r = 0.48, p< 0.05). In contrast, leaf area under high R/FR was negatively correlated with SPAD under low R/FR (r = -0.47, p< 0.05) (**Table 2**).

**Table 2:** Cross-environment correlations among seedling traits measured under high (control) and low (shade) R/FR ratios.

High R/FR ratio (control)	Low R/FR ratio (shade)					
	Plant height (cm)	Leaf area (cm^2^)	Stem (stalk) diameter (mm)	Root length (cm)	SPAD index	Dry biomass (g)
Plant height (cm)	0.62***	-0.12	0.39*	0.13	0.18	0.53**
Leaf area (cm^2^)	-0.26	0.96***	0.08	0.36	-0.47*	-0.07
Stem (stalk) diameter (mm)	0.26	0.06	0.59**	-0.08	0.06	0.63***
Root length (cm)	0.24	0.48*	0.07	0.88***	-0.34	-0.08
SPAD index	0.19	-0.19	0.13	-0.37	0.72***	0.38
Dry biomass (g)	0.25	0.18	0.43*	-0.15	0.10	0.59**

Pearson correlation coefficients (r) were calculated between seedling traits measured under high R/FR (rows) and low R/FR (columns). Traits included plant height, leaf area, stem diameter, root length, SPAD, and dry biomass. Values on the diagonal indicate correlations for the same trait across the two light environments, whereas off-diagonal cells indicate correlations between different traits across environments.

Numbers inside table cell are correlation values, and significance is indicated as *p < 0.05, **p < 0.01, ***p < 0.001, absence/ns denotes non-significant correlations (p > 0.05).

### 3.3 Seedling plant architecture traits

Most seedling traits changed across 100 years of maize breeding (**Supplementary Table 2**), but the overall magnitude of shade response did not show a directional historical trend.

Across the 102-year release interval (1920-2022), percentage changes indicated marked shifts in seedling architecture, while trends were generally similar under low and high R/FR ratios. Leaf areas of fully expanded second leaves declined significantly over time under both low and high R/FR, by 0.066 cm^2^·yr^−1^ under low R/FR and 0.067 cm^2^·yr^−1^ under high R/FR, corresponding to reductions of 26.7% and 28.3%, respectively, over the 1920–2022 interval (p< 0.01). The estimated differences in slopes between light treatments were small (total difference of 1.6%) and not statistically significant. Root lengths declined across release years, decreasing by decreasing by 0.020 cm·yr^−1^ under low R/FR and 0.024 cm·yr^−1^ under high R/FR (reductions of 9.8% and 11.4%, respectively, from 1920 to 2022). This temporal decline was statistically significant under high R/FR (p< 0.01), whereas this trend was not significant at low R/FR.

SPAD indexes significantly increased over time under both low and high R/FR, by 0.037 and 0.029 SPAD units·yr^−1^ under low and high R/FR, corresponding to increases of 11.3% and 9.1% from 1920 to 2022 (p< 0.01). The difference between light treatments was small (1.81%) and not significant. Temporal trends for plant height, stem (stalk) diameter, and dry biomass were not statistically significant (**Figure 1**).

The root length/plant-shoot height ratio fell by 17.5% under high R/FR and 13.3% under low R/FR (p< 0.01) in seedlings, indicating a shift toward shorter seedling root systems relative to shoot height in modern hybrids. Moreover, the plant height/stem (stalk) diameter ratio increased by 11.1-9.9% (p< 0.05), reflecting taller, more slender seedlings (**Figure 1**).

Multivariate analysis nevertheless confirmed a strong directional response to low R/FR across the six seedling traits (Hotelling’s T^2^ = 701.52, F_6,21_ = 94.44, p<0.001). However, the principal component analysis (PCA) displacement length for six seedling traits, measured as Euclidean distance, analysis showed no significant trend in shade-response magnitude across genotype release years (β = 0.0042 ± 0.0047 shift units·year^−1^, p = 0.488, R^2^ = 0.04). A Spearman rank test indicated a small, non-significant association (ρ = 0.172, p = 0.390; n = 27 genotypes). Shade-response traits in maize remained stable over the past century, with no changes in plasticity variation (**Figure 1**).

### 3.4 Effect of low R/FR light conditions on stomatal traits and long-term trends

We observed an increase in stomatal density, but a decrease in stomatal size in relation to the release year of the genotype for low R/FR ratios. The total stomatal pore area was negatively correlated with the year of release 1920 to 2022 (r = -0.51, p< 0.05) (**Figure 2**). Notably, these responses were broadly similar to those observed previously under a high R/FR ratio (Bilgici et al., 2026), supporting the consistency of the stomatal trend across experiments. Under low R/FR, release year was significantly associated with smaller stomatal size (r= -0.46, p< 0.01), and lower total stomatal pore area (r = -0.51, p< 0.05). Under high R/FR, release year was significantly associated with higher stomatal density (r = 0.46, p< 0.01), and lower total stomatal pore area (r = -0.45, p< 0.01) (Bilgici et al., 2026).

**Figure 2:**
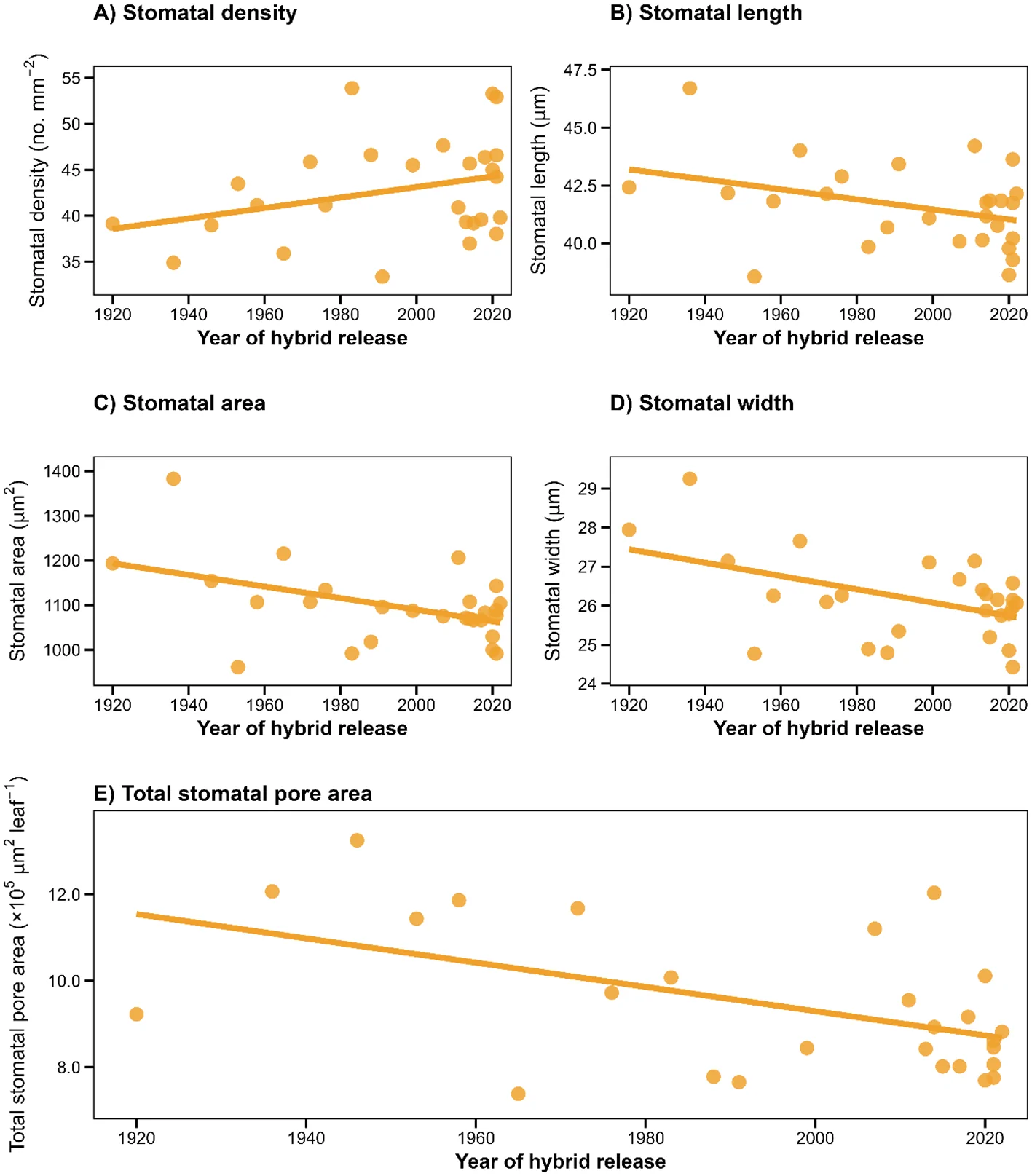
Relationships between maize genotype release year (1920-2022) and stomatal traits under Low R/FR. Panels show **(A)** stomatal density (per mm^2^), **(B)** stomatal length (µm), **(C)** stomatal size (area) (µm^2^), **(D)** stomatal width (µm), and **(E)** total stomatal pore area (µm^2^). Points represent genotype values; solid lines show fitted linear trends.

### 3.5 Correlations of stomatal, morphological, and physiological traits for low R/FR ratio

Under low (shade) R/FR (**Table 3**) stomatal density was closely negative correlated with plant height, leaf area, stem (stalk) diameter, root length and dry biomass. Seedling traits were also closely correlated (plant height-dry biomass r = 0.80; plant height-stem (stalk) diameter r = 0.77; stem (stalk) diameter-dry biomass r = 0.81), with SPAD showing moderate positive associations with height (r = 0.30-0.32). Importantly, stomatal traits were coupled to seedling performance through a consistent trade-off: greater plant height was associated with lower stomatal density and smaller stomata (plant height-stomatal density r = -0.55; plant height-stomatal width r = -0.58; plant height-stomatal size r = -0.53). Total stomatal pore area tracked positively with leaf area (r = 0.62), negatively with stomatal density (r = -0.16), but was negatively related to vigor (plant height r = -0.60; dry biomass r = -0.49; stem (stalk) diameter r = -0.48).

**Table 3:** Pearson correlations between seedling traits and stomatal traits under low R/FR (shade).

Trait	Stomatal density (mm^2^)	Stomatal size (um^2^)	Stomatal length (um)	Stomatal width (um)	Total stomatal pore area (um^2^)
Plant height (cm)	-0.55***	-0.53***	-0.52	-0.58***	-0.60***
Leaf area (cm^2^)	-0.16*	0.12	0.10	0.11	0.62***
Stem (stalk) diameter (mm)	-0.57***	-0.35***	-0.37***	-0.42***	-0.48***
Root length (cm)	-0.16*	0.10	0.04	0.03	0.01
SPAD index	-0.11	-0.11	-0.12	-0.14	-0.19**
Dry biomass (g)	-0.54**	-0.42	-0.42***	-0.47***	-0.49***

Pearson correlation coefficients (r). Asterisks indicate significance: *p< 0.05, **p< 0.01, ***p< 0.001; n.s., not significant.

Cross-light condition correlation analysis showed that the same stomatal traits measured under high (control) and low (shade) R/FR ratios were positively and significantly correlated, including stomatal density (r = 0.75, p< 0.001), stomatal size (r = 0.71, p< 0.001), stomatal length (r = 0.79, p< 0.001), stomatal width (r = 0.48, p< 0.01), and total stomatal pore area (r = 0.82, p< 0.001). These results indicate that genotype-specific trait differences were largely maintained across light conditions (**Table 4**).

**Table 4:** Pearson correlation between stomatal traits under low (shade) and high (control) R/FR ratios.

	Low R/FR ratio (shade)				
	Stomatal density (mm^2^)	Stomatal size (um^2^)	Stomatal length (um)	Stomatal width (um)	Total stomatal pore area (um^2^)
High R/FR ratio (control)					
Stomatal density (mm^2^)	0.75***	-0.55**	-0.53**	-0.49	-0.19
Stomatal size (um^2^)	-0.58**	0.71***	0.77***	0.50**	0.05
Stomatal length (um)	-0.51***	0.67**	0.79***	0.42*	0.07
Stomatal width (um)	-0.53 **	0.57 **	0.55**	0.48*	-0.04
Total Stomatal Pore Area (um^2^)	-0.10	0.08	0.05	0.07	0.82***

Pearson correlation coefficients (r). Asterisks indicate significance: *p< 0.05, **p< 0.01, ***p< 0.001; n.s., not significant.

## 4 Discussion

### 4.1 Plant architecture traits changed over 100 years of maize breeding at the seedling stage

Managing plant-to-plant competition is a fundamental challenge for increasing maize production without expanding to non-agricultural lands or introducing new environmentally damaging production practices. Our central question was whether long-term breeding for higher yield by increasing planting densities inadvertently altered maize response to shade at the seedling stage, due to its importance for PGC systems. We tested the hypothesis that recent hybrids would show seedling traits consistent with adaptation to denser, more competitive canopies, including altered shoot architecture, modified root allocation and reduced shade-avoidance response under low (shade) R/FR ratios.

We found that modern hybrids differed from older ones in several traits at the seedling stage, but the magnitude of their response to low (shade) R/FR ratio did not show a clear trend across release years. Instead, long-term breeding appeared to have altered baseline seedling architecture. A century of yield-directed selection had a clear and consistent impact on the several key aspects of morphology of maize seedlings. Under both low (shade) and high (control) R/FR ratios conditions, modern hybrids had smaller leaf areas, shorter root lengths, and higher SPAD indexes compared to historical hybrids (**Supplementary Figures 3**, **5**). The decline in root length to shoot height ratio with year of release further suggests that long-term breeding has modified early shoot-root allocation. Newer hybrids tended to have proportionally less root length relative to shoot height at the seedling stage. Genotype x light condition interactions were not significant, but this should not be interpreted as evidence that the estimated genotype × light variance component was identically zero; these architectural changes reflect baseline trait shifts rather than breeding-associated change in low R/FR (shade-response plasticity).

Under the field conditions, ERA-hybrid studies showed that long-term maize breeding for high grain yield has resulted in indirect changes in traits such as root architecture (York et al., 2015), improved radiation (Messina et al., 2022), water (Rotundo et al., 2025), and nitrogen use efficiency (RUE, WUE, NUE) (DeBruin et al., 2017). Moreover, per-plant grain yield (Borras et al., 2026) and drought tolerance increased. Other phenotypes include smaller tassels, steeper leaf angle, stay-green (longer vegetative duration) (Lee and Tollenaar, 2007), as well as tolerance to high planting densities. Similarly, Bayer legacy hybrid studies conducted at the mature stage indicate that modern maize breeding successfully enhanced both yield potential and drought-stress tolerance. The observed yield gains were primarily attributed to increased kernel numbers, a reduced anthesis-silking interval, and improved WUE (Danalatos et al., 2026). Additionally, changes related to leaf angle (Elli et al., 2023), specific leaf area (Kalogeropoulos et al., 2024), root depth (Sciarresi et al., 2024), and harvest index (Ruiz et al., 2023) contributed to these outcomes. However, faster and deeper root growth was not always beneficial for achieving higher yields (Sciarresi et al., 2024).

However, to our knowledge, this is the first study to investigate the effects of 100 years of long-term breeding on seedling morphology and physiology in response to both low and high R/FR ratios. Despite extensive studies on maize shade responses, no study has directly compared historical versus modern hybrids under controlled low R/FR conditions during seedling establishment. Prior studies emphasize adult canopy traits, flowering-stage responses, or model systems (Dubois et al., 2010; Wang et al., 2016; Wu et al., 2019; Jafari et al., 2024; Escobar‐Bravo et al., 2024) in the short-term assessments (Islam, 2023). Seedling-stage responses to long-term breeding over several eras are poorly understood.

Our observation of a declining leaf area is consistent with findings from a study (Kalogeropoulos et al., 2024) conducted between 1983 and 2017, which demonstrated a clear and consistent trend during the mature phase. Similarly, our findings regarding decreased root length align with results from studies (York et al., 2015; Rinehart et al., 2024) on mature-stage root length and seedlings derived from hybrids released over 80 years. Additionally, the increased SPAD index observed in our study is consistent with greater leaf chlorophyll status and may indicate increased specific leaf nitrogen (SLN; g N m^-2^ leaf area). This interpretation aligns with DeBruin et al. (2017), who reported that greater N supply increased SLN in U.S. Midwest maize hybrids released between 1934 and 2013, alongside differences in N accumulation and remobilization patterns associated with genetic improvement and grain yield. Our measurement is of the longest root of a seedling grown, whereas the field studies cited describe nodal root systems in mature plants. Seminal and nodal roots differ in origin, in the timing of their growth and in how they interact with soil structure and water distribution. The same caveat applies to the comparisons with mature-stage leaf area and canopy traits discussed above. We found no significant trends in plant height, which aligns with previous studies (Rinehart et al., 2024). A hydroponic study involving historical hybrids from 1930 to 2000 indicated that there was no significant change in the root-to-shoot ratio of the seedlings (Sanguineti et al., 2006). However, it also noted a notable trend of smaller size and weight in the seedlings (both roots and shoots) of newer hybrids compared to those developed in earlier years. This restrained growth during the seedling phase in newer hybrids was an unexpected finding for the researchers.

Our study’s findings align with the agronomic pressures experienced over this century, rather than with any deliberate manipulation of seedling shade responses. Importantly, the light treatment did not affect the overall trends observed (no significant genotype × treatment interactions), indicating that breeding practices altered the baseline architecture of the seedlings instead of their light-quality plasticity (**Table 1** and **Figure 1**). The lack of significant genotype x light quality interactions suggests similar responses of genotypes with very different year of release to high and low R/FR ratios in this study. The response was largely uniform across genotypes: when exposed to low R/FR, many of genotypes elongated, reduced stem (stalk) diameter, and decreased root investment in similar proportions. Our results provide evidence for a strong multivariate seedling response to low R/FR but do not provide evidence that its magnitude changed systematically during the breeding period represented by these hybrids. Within the traits, developmental stage, environmental conditions, and statistical precision examined here, we found no detectable evidence that long-term maize breeding for yield systematically altered the overall magnitude of the seedling response to low R/FR. What did change over breeding time were the traits means (leaf area, root length, and SPAD) themselves the starting points upon which the conserved SAR program operates rather than the magnitude or sensitivity of that long-term breeding program. In our study, second-leaf area, root length, and SPAD index increased under low R/FR, whereas these traits may decline when spectral shade is accompanied by reduced irradiance. Importantly, PPFD was maintained at 743 µmol m^-2^ s^-1^ in both chambers; thus, the treatments primarily contrasted spectral quality rather than the combined spectral and irradiance changes typical of canopy shade. Under natural canopy shade or neutral-density shading, low R/FR commonly occurs together with lower photosynthetic photon flux and may constrain carbon availability, contributing to reduced leaf expansion, thinner leaves, and lower chlorophyll status. By contrast, under our equal-PPFD conditions, the far-red signal was experimentally decoupled from a reduction in 400–700 nm photon flux. This allowed low R/FR-mediated developmental responses, including enhanced organ elongation and increased leaf expansion, to be expressed without the confounding effects of reduced PPFD.

### 4.2 High-density tolerance may not necessarily confer readiness for perennial ground cover systems

Modern high-yielding maize hybrids thrive at much higher planting densities than older varieties (Duvick, 2005b). As maize planting densities increased 3.6-fold over the last century (USDA-NASS, 2025), we expected that modern hybrids would show increased ST. Plants use two main strategies to adapt to low (R/FR) light conditions: SAR and ST (Maddonni et al., 2002; Page et al., 2010). In maize, SAR occurs during early growth when R/FR ratios drop due to stem elongation and changes in resource allocation, often triggered by competition (Hu et al., 2023; Yan et al., 2024; Moro et al., 2026). Conversely, ST allows maize to thrive in low-light without excessive elongation, maintaining compact structures and optimizing light capture while preserving photosynthetic capacity (Collison et al., 2020; Casal and Fankhauser, 2023; Menalled et al., 2024). Understanding the difference between SAR and ST is essential, especially in how maize seedlings respond to light in conventional versus PGC or intercropping systems. In situations of strong competition, maize with low SAR may be better at minimizing yield losses. In our study, heritabilities (H^2^) for maize seedling traits ranged from 0.54 to 0.96, with higher values under a high R/FR ratio (H^2^ = 0.66-0.96) compared to a low R/FR ratio (H^2^ = 0.54-0.86) (**Table 1**). Lower heritabilities under low R/FR conditions suggest increased environmental variability, making trait selection less reliable. High R/FR conditions promote clearer expression of genetic differences among genotypes. Shade responses can alter early seedling allometry (the plant shifting its energy allocation); therefore, they might prioritize stem/internode elongation at the expense of leaf expansion, or alter the shoot-to-root ratio, and remain an important component of early maize seedling and canopy plasticity.

The absence of a release-year trend in shade-response magnitude does not mean that shade responsiveness is fixed. Genetic variation for shade response is still present in modern germplasm. Traits indicating SAR include increased plant height, reduced stem diameter, decreased leaf area, and shifts in root or biomass allocation under low R/FR conditions, which can be seen as an “escape” strategy. In contrast, ST characterized as “endurance” maintains leaf area, exhibits less excessive stem elongation, and stabilizes biomass accumulation without aggressive upward growth under low R/FR or with minimal negative changes. Our findings suggest that some modern hybrids exhibited stronger SAR-like responses, particularly increased elongation under low R/FR, but this pattern was not consistent across all modern hybrids or strongly associated with year of release compared to older hybrids. In contrast, hybrids released earlier in the historic hybrid ERA can be considered possible ST candidates or weak SAR. However, long-term breeding changed trait distributions (**Figure 1**). While some modern hybrids demonstrate strong SAR, there is no clear trend indicating that modern hybrids, as a group, show an overall increase or decrease in shade response. Therefore, this suggests that shade response behavior may be more specific to individual hybrids rather than related to the year of release.

Our findings indicate that useful variation for SAR and ST exists within modern maize germplasm and is not primarily explained by year of release. This suggests that adaptation to PGC systems should be pursued through targeted screening of specific individual lines or hybrids for seedling traits that maintain growth, biomass, and physiological function under low R/FR conditions. By stability we mean performance stability, that is, the maintenance of leaf area, biomass accumulation and SPAD index when the R/FR ratio falls, rather than the absence of plasticity as such. A genotype that elongates strongly under low R/FR is highly responsive, but in a PGC system that responsiveness is a cost: it diverts resources into stem extension, reduces stem diameter and root growth, and increases lodging risk without any compensating gain, because the PGC cannot be overtopped and outcompeted in the way a neighbouring maize plant can. The screening target for PGC is therefore a genotype that maintains function under a persistent far-red-enriched signal, not the most responsive genotype. Thus, the breeding opportunity lies not in returning to older germplasm, but in developing rapid phenotyping pipelines to identify and select shade-resilient maize ideotypes for PGC-based systems.

### 4.3 Stomatal traits under low R/FR (shade) conditions changed over the past 100 years

Our study of stomatal traits (**Supplementary Figures 3**, **6**) focusing on the second leaf, our previous study (Bilgici et al., 2026) showed that measurements from the first leaf reliably predicted characteristics of the ear leaf. Stomatal density increased while stomatal size decreased when comparing historical and modern hybrids at a low R/FR ratio. In addition, modern hybrids exhibited a reduction in total stomatal pore area relative to historic hybrids (**Figure 2**, **Table 3**). These responses were similar to those observed in the study using a high R/FR ratio (Bilgici et al., 2026), supporting the consistency of the stomatal trend across experiments. This may have been indirectly selected in modern breeding programs (Lee and Tollenaar, 2007). The decline in total stomatal pore area with release year parallels the trends observed for leaf area and root length, suggesting a broader shift toward more conservative early-season resource allocation in modern hybrids. However, it remains unclear whether the reduction in total stomatal pore area reflects direct selection for improved WUE, an indirect consequence of selection for reduced leaf area, or selection for other traits. Since 1934 (Rotundo et al., 2025), U.S. corn-belt maize rainfed yields increased 1.9%/year^-1^. The observation that modern hybrids exposed to low R/FR express a smaller total stomatal pore area than historic hybrids adds a functional dimension to the morphological characterization. Additionally, stomatal density had a negative relationship, while stomatal size showed a positive relationship with atmospheric CO_2_ concentration and temperature anomaly (°C) over the past century (Bilgici et al., 2026).

### 4.4 Implications for maize breeding for PGC systems

A century of yield-focused breeding reshaped maize seedling architecture producing taller, narrower leaf area, shorter roots, and more chlorophyll-dense plants as an indirect consequence of selecting for high yield under high planting densities. We speculated that this process might have simultaneously enhanced shade tolerance, yielding germplasm pre-adapted to PGC systems as an unintended consequence. We found a constitutive shift in allometry (changed architecture such as increased SPAD, decreased leaf area and root length) rather than a change in phenotypic plasticity. Modern hybrids are driven by high shoot investment rather than a change in shade sensitivity. Our findings indicate that modern hybrids have not evolved different shade reactions. They displayed a different baseline architecture. The overall shade-response magnitude did not change significantly over release year (**Figure 1**).

Density tolerance may therefore not translate directly to PGC readiness. These findings support developing a dedicated PGC-tolerance ideotype that combines moderate SAR with stronger ST. The ideal seedling should establish faster under competition, but avoid excessive elongation under low R/FR because strong SAR may increase height while reducing stem diameter, root growth, and lodging resistance. For future maize breeding, priority traits for PGC breeding should include early vigor, moderated elongation, thicker basal stems, stable leaf area and biomass, maintained SPAD, vigorous rooting, and lodging resistance. Deep root or root length is important because established PGC compete strongly in the upper soil layer for water and nutrients. This ideotype may partially contrast with the trajectory of modern high-density maize breeding, which has favored compact canopy architecture and density tolerance, because PGC systems require tolerance to earlier and more asymmetric above- and belowground competition. The observed genetic variation among hybrids for height, stem diameter, root length, leaf area, biomass, and physiological traits suggests that these traits are available for selection. Translating these mechanistic insights into actionable strategy would require linking seedling-stage phenotypes to field performance under PGC competition.

A limitation of this study is that each R/FR treatment was maintained in a single dedicated growth chamber across all temporal runs. Chamber identity was therefore confounded with light treatment, and chamber-specific variation cannot be fully separated from the effect attributed to R/FR. Plants were randomized and repositioned within chambers, and chamber environmental conditions were monitored throughout, but these procedures address within-chamber heterogeneity rather than providing chamber-level replication. The increases in leaf area and root length under low R/FR may reflect the non-limiting resource conditions of this experiment. Unlike true shade, where reduced PAR imposes carbon limitation and may favor allocation to stem elongation, low R/FR alone can induce elongation responses through phytochrome B-PIF-auxin signaling. Thus, these responses likely represent low-R/FR-specific plasticity rather than compensatory allocation; field responses may differ when low R/FR is accompanied by reduced PAR (Fernández-Milmanda and Ballaré, 2021). Independent field data nonetheless supports the biological relevance of these responses. A subset of the hybrids evaluated here was also tested in three years of field trials comparing conventional maize production with a Kentucky bluegrass PGC system (Bilgici et al., in preparation). Pairing low R/FR chamber treatment with the PGC system and the high R/FR control with conventional management, hybrid rankings for seedling height, SPAD, and dry biomass in the chamber were closely associated with rankings for the corresponding mature-stage traits in the field (ρ = 0.9; P-value< 0.001). This is informative because chamber confounding primarily limits inference about the treatment main effect, whereas these rankings reflect genotypic variation expressed within a common chamber environment; their agreement with independent field trials indicates that seedling responses capture field-relevant genotypic variation rather than a chamber artifact. Two caveats remain. The chamber treatment isolates only the light-quality component of interspecific competition, whereas Kentucky bluegrass also competes with maize for water and nutrients and alters soil temperature and below-ground conditions, so field testing remains essential for biological validation. We emphasize that all inferences here are restricted to the seedling stage. Seedling-stage responses to neighbor-derived far-red signals are mechanistically distinct from the canopy-level crowding tolerance that underlies density adaptation at flowering and grain fill, and our hypothesis is that selection under increasing density has indirectly altered the seedling response. Independent field validation would therefore be important for determining whether the genotypic patterns observed at the seedling stage translate to agronomically relevant competitive environments. In addition, the design does not permit formal separation of chamber and treatment effects; future experiments should rotate R/FR treatments among chambers across runs or replicate each treatment across multiple chambers. Within these constraints, our findings support controlled low R/FR phenotyping as a rapid early-stage screen for maize hybrids adapted to PGC systems.

## Data Availability

The datasets presented in this study can be found in online repositories. The names of the repository/repositories and accession number(s) can be found in the article/**Supplementary Material**.
