# Supplementary material for: Shade avoidance responses in seedlings of maize hybrids representing 100 years of breeding history
Source: Front Plant Sci. 2026 Sep 30;17:1939000. doi: 10.3389/fpls.2026.1939000 (PMC13662160; doi:10.3389/fpls.2026.1939000)
Supplement: Supplementary file 1 [file DataSheet1.docx]

**Additional Supplementary Data**

**Shade avoidance responses in seedlings of maize hybrids representing 100 years of breeding history**

**Authors:** Memiş Bilgici^1^*, Sara Lira^2^, Lucas Borras^2^, Elnaz Ebrahimi^1^, Kenneth J Moore^1^, D. Raj Raman^3^, Thomas Lübberstedt^1^

**Affiliations:**

^1^Department of Agronomy, Iowa State University, Ames, IA, 50011-1051, USA

^2^Corteva Agriscience, 7200 NW Ave., Johnston, IA, 50310, USA.

^3^Department of Agricultural and Biosystems Engineering, Iowa State University, Ames, IA 50011, USA

Corresponding Author: Memiş Bilgici^1^*

Email: [bilgici@iastate.edu](mailto:bilgici@iastate.edu)

Supplementary Figure 1. Schematic representation of PGC crop canopy on maize seedling growth as affected by light in Maize-PGC system. Light, nutrients, water, and weed competition between the maize seedling stage and existing PGC.

Supplementary Figure 2. The left image shows a maize seedling planted in a PGC real field, while the right image displays the maize at the V7-8 stage.

Supplementary Figure 3. Illustration of the effects of low and high R/FR ratios on maize morphological, physiological and stomata responses.

Supplementary Figure 4. Illustration of plant growth, affected by low R/FR ratio light (left) and Control light (right), plant height (elongation) is high and no or less anthocyanins color on the stem under low red far-red ratio light.

Supplementary Figure 5. Comparison of plant traits for 27 maize genotypes under low and high R/FR ratios.

Supplementary Figure 5 shows a comparison of plant traits for 27 maize genotypes under low and high R/FR ratios. The changes relative to the high R/FR control were as follows: plant height increased by 4.48 cm, stem diameter decreased by 0.03 mm, leaf area increased by 1.16 cm², root length increased by 0.51 cm and the SPAD index increased by 1.14 units. All of these changes were statistically significant (p < 0.01). Dry biomass differed by 0.01 g and was not significant.

Supplementary Figure 6. 40X microscopic magnification of stomata under low R/FR (left) and high R/FR (right) light.

Supplementary Figure 7. Correlations of traits for 27 maize genotypes under low and high R/FR ratios.

Under high R/FR, plant height was positively correlated with stem diameter (r = 0.64) and dry biomass (r = 0.69), while stem diameter was very closely associated with dry biomass (r = 0.86). Plant height and dry biomass were negatively related to the root length/height ratio (r = -0.73 and -0.69, respectively). Under low R/FR, these relationships generally remained but were somewhat weaker; such as, stem diameter-dry biomass decreased to r = 0.75 and plant height-root length/height to r = -0.47. Low R/FR also strengthened some root-related relationships. Root length was closely positively correlated with root length/height (r = 0.70), and leaf area became positively associated with root length/height (r = 0.53). SPAD was negatively related to root length/height under both conditions, with the relationship slightly stronger under low R/FR (r = -0.52).

Supplementary Figure 8. Traits of maize plants from historic genotypes and modern genotypes under low and high R/FR ratios.

The supplementary figure 8 compares the traits of maize plants from historic ERA genotypes and modern hybrids under low and high R/FR ratios. Root length (cm) significantly decreased in modern hybrids under both high R/FR and low R/FR conditions, with reductions of -2.51 cm (p < 0.001) for high R:FR and -2.09 cm (p < 0.001) for low R/FR. SPAD index (%), significantly increased in modern hybrids under both conditions. The increase was +2.32 % (p < 0.01) under high R/FR and +1.80 % (p < 0.05) under low R/FR. Leaf area decreased significantly under low R/FR conditions, with a reduction of -2.67 cm² (p < 0.05) and -2.6cm² (p < 0.05) under high R/FR conditions. Plant height (cm) under low R/FR ratio, historic ERA genotypes measured an average of 31.0 cm, while modern hybrids measured 30.8 cm, resulting in a reduction of 0.1 cm, although this change is not significant. In contrast, under high R/FR ratios, historic era genotypes were 25.9 cm tall compared to 26.9 cm in modern hybrids, indicating an increase of 1 cm, which is also not significant. Stem diameter (mm) for historic ERA hybrids, the average stem diameter was 0.47 mm under low R/FR conditions, compared to 0.46 mm for modern hybrids, showing an increase of 0.01 mm that is not significant. Under high R/FR conditions, historic ERA genotypes had an average diameter of 0.49 mm, while modern hybrids measured 0.50 mm, reflecting an increase of 0.01 mm that is not significant. Dry biomass (g) under low R/FR conditions, historic ERA genotypes had an average dry biomass of 0.28 g, while modern hybrids showed an increase to 0.29 g, resulting in a 0.01 g increase. Under high R/FR conditions, historic ERA genotypes measured 0.26 g compared to modern hybrids at 0.30 g, showing an increase of 0.04 g; however, all changes observed are not significant.

Supplementary Figure 9 presents the performance ranking of 27 maize genotypes across all traits and light conditions (high R/FR vs. low R/FR).

The supplementary figure 9 presents the performance ranking of 27 maize genotypes across all traits and light conditions (high R/FR vs. low R/FR). Each line represents one specific hybrid across the six different traits. The color coding indicates the performance ranking within each trait-environment combination. Additionally, the line patterns illustrate hybrid-by-light interactions, although not all of these interactions are statistically significant. Although each genotype showed variation in the direction and magnitude of trait responses between high R/FR and low R/FR conditions, hybrids that perform well in high R/FR also maintain high performance in low R/FR conditions.

Supplementary Figure 10. Principal component analysis of maize seedling traits under high and low R/FR conditions.

The supplementary figure 10 shows that six traits such as plant height, leaf area, stem diameter, root length, SPAD, and dry biomass were standardized prior to principal component analysis (PCA). Points represent model-derived genotype × light-condition estimates, with labels indicating hybrid year of release. Circles represent high R/FR and triangles represent low R/FR conditions. Historical hybrids (1920-2012) are shown in blue and modern hybrids (2013-2022) in orange. Lines connect observations for the same genotype, illustrating the multivariate shift from high to low R/FR. Black vectors represent trait loadings projected into PCA score space. PC1 and PC2 explained 38.9% and 24.5% of the total multivariate variation, respectively, accounting for 63.4% of the total variance. Light treatment produced visible within-genotype shifts in multivariate trait space, whereas the direction and magnitude of these shifts showed no clear separation between historical and modern breeding eras. Across genotypes, the changes relative to the high R/FR control were as follows: plant height increased by 4.48 cm, stem diameter decreased by 0.03 mm, leaf area increased by 1.16 cm², root length increased by 0.51 cm and the SPAD index increased by 1.14 (%). All of these changes were statistically significant (p < 0.01). Dry biomass differed by 0.01 g and was not significant.

Supplementary Table 1. genotype and light effects over measured traits in 14 days second leaf

|  | Treatment | Plant height (cm) | | Leaf area (cm^2^) | | Stem (Stalk) diameter (mm) | | Root length (cm) | | SPAD index | | Dry biomass  (g plant^-1^) | |
| --- | --- | --- | --- | --- | --- | --- | --- | --- | --- | --- | --- | --- | --- |
|  | Genotype | High  R/FR | Low R/FR | High R/FR | Low R/FR | High  R/FR | Low R/FR | High R/FR | Low R/FR | High R/FR | Low R/FR | High R/FR | Low R/FR |
| Historic ERA  Genotypes | Reid YD | 25.9 | 30.8 | 20.1 | 19.1 | 0.54 | 0.51 | 18.4 | 18.5 | 32.9 | 33.3 | 0.29 | 0.28 |
|  | 307 | 23.4 | 28.4 | 22.5 | 25.0 | 0.51 | 0.47 | 18.7 | 19.4 | 37.1 | 37.2 | 0.27 | 0.29 |
|  | 352 | 26.6 | 30.4 | 29.0 | 29.1 | 0.54 | 0.49 | 21.3 | 21.5 | 36.4 | 35.3 | 0.35 | 0.32 |
|  | 354HYB | 25.2 | 29.1 | 26.9 | 27.3 | 0.46 | 0.43 | 21.1 | 21.3 | 32.2 | 31.8 | 0.24 | 0.24 |
|  | 354A | 23.1 | 29.2 | 22.8 | 26.0 | 0.44 | 0.43 | 22.2 | 21.3 | 31.5 | 32.5 | 0.25 | 0.25 |
|  | 3376 | 24.6 | 31.2 | 16.1 | 16.9 | 0.50 | 0.50 | 18.8 | 19.3 | 34.6 | 35.4 | 0.24 | 0.30 |
|  | 3366 | 26.1 | 32.0 | 20.3 | 23.1 | 0.47 | 0.48 | 21.6 | 20.4 | 35.0 | 35.3 | 0.25 | 0.30 |
|  | 3382 | 24.3 | 29.4 | 21.1 | 20.7 | 0.47 | 0.49 | 19.4 | 18.8 | 33.6 | 36.7 | 0.26 | 0.26 |
|  | 3378 | 21.7 | 30.0 | 18.7 | 19.8 | 0.41 | 0.45 | 18.6 | 20.6 | 28.3 | 34.8 | 0.17 | 0.22 |
|  | 3379 | 28.4 | 33.2 | 14.9 | 16.5 | 0.54 | 0.49 | 18.8 | 18.6 | 37.1 | 36.9 | 0.29 | 0.37 |
|  | 3394 | 32.1 | 35.4 | 20.8 | 20.7 | 0.53 | 0.50 | 20.8 | 22.5 | 33.7 | 34.9 | 0.32 | 0.33 |
|  | 33P67 | 28.2 | 31.5 | 16.0 | 16.6 | 0.48 | 0.46 | 21.4 | 23.1 | 32.7 | 36.9 | 0.25 | 0.26 |
|  | 33T59 | 26.8 | 32.4 | 19.2 | 22.0 | 0.49 | 0.47 | 23.2 | 22.8 | 35.8 | 36.6 | 0.26 | 0.28 |
|  | P1151 | 26.6 | 31.3 | 18.8 | 19.2 | 0.50 | 0.48 | 21.2 | 22.0 | 35.0 | 38.1 | 0.26 | 0.27 |
| Modern hybrids | P1197A | 29.8 | 32.9 | 20.3 | 20.2 | 0.54 | 0.49 | 18.3 | 19.0 | 36.7 | 38.1 | 0.35 | 0.35 |
|  | P0574 | 28.3 | 30.3 | 22.9 | 27.0 | 0.53 | 0.47 | 17.5 | 18.0 | 36.9 | 37.6 | 0.32 | 0.30 |
|  | P1197B | 28.4 | 29.0 | 20.6 | 21.7 | 0.52 | 0.48 | 17.8 | 19.2 | 35.1 | 37.3 | 0.33 | 0.32 |
|  | P1185 | 26.1 | 27.2 | 18.4 | 19.2 | 0.55 | 0.45 | 17.4 | 17.7 | 34.5 | 35.3 | 0.33 | 0.25 |
|  | P1366 | 25.6 | 28.7 | 17.7 | 18.7 | 0.50 | 0.46 | 16.8 | 19.3 | 34.1 | 35.6 | 0.34 | 0.29 |
|  | P1244 | 25.5 | 30.5 | 17.4 | 18.2 | 0.49 | 0.44 | 17.6 | 18.9 | 34.1 | 35.7 | 0.32 | 0.27 |
|  | P1587 | 21.7 | 30.2 | 18.4 | 20.0 | 0.44 | 0.47 | 18.4 | 20.4 | 37.5 | 39.7 | 0.22 | 0.28 |
|  | P1548 | 22.1 | 27.9 | 15.8 | 16.6 | 0.43 | 0.43 | 18.0 | 17.7 | 37.7 | 37.4 | 0.20 | 0.24 |
|  | P0953 | 29.5 | 28.8 | 17.8 | 18.4 | 0.51 | 0.43 | 17.3 | 19.2 | 37.1 | 36.5 | 0.32 | 0.23 |
|  | P0995 | 26.4 | 33.4 | 14.0 | 15.9 | 0.46 | 0.45 | 18.8 | 19.9 | 39.8 | 38.6 | 0.25 | 0.27 |
|  | P1082 | 26.3 | 30.6 | 15.0 | 16.1 | 0.49 | 0.46 | 17.5 | 17.8 | 36.5 | 37.0 | 0.28 | 0.29 |
|  | P1222 | 30.4 | 34.7 | 16.2 | 16.7 | 0.50 | 0.47 | 18.9 | 19.3 | 36.7 | 38.0 | 0.31 | 0.30 |
|  | P1413 | 28.8 | 36.6 | 17.7 | 19.6 | 0.54 | 0.51 | 18.6 | 20.7 | 35.8 | 37.0 | 0.35 | 0.39 |
|  |  |  |  |  |  |  |  |  |  |  |  |  |  |
| Environment | High vs Low R/FR | 26.5 | 30.9 | 19.3 | 20.4 | 0.50 | 0.47 | 19.4 | 19.9 | 35.1 | 36.2 | 0.28 | 0.29 |
| Genotype |  | ** | | ** | | ** | | ** | | ** | | ** | |
| Environment |  | ** | | ** | | ** | | ** | | ** | | ns | |
| GxE |  | ns | | ns | | ns | | ns | | ns | | ns | |

Asterisks indicate significance: * p < 0.05, ** p < 0.01, *** p < 0.001; n.s., not significant (p > 0.05).

Supplementary Table 2. Long-term changes in seedling traits under high and low R/FR. Slopes are per year; percentage change is expressed relative to the fitted 1920 value over the 102-year interval.

| Trait | Light | r | Slope per year | p-value | Absolute change | Change (%) |
| --- | --- | --- | --- | --- | --- | --- |
| Plant height | High R/FR | 0.41 | 0.0201 | 0.34 | 2.052 | +8.2 |
|  | Low R/FR | 0.17 | 0.0073 | 0.73 | 0.745 | +2.5 |
| Stem diameter | High R/FR | -0.16 | -1.27e-04 | 0.65 | -0.013 | -2.6 |
|  | Low R/FR | -0.45 | -3.07e-04 | 0.27 | -0.031 | -6.4 |
| Leaf area | High R/FR | -0.60 | -0.0671 | <0.01 | -6.842 | -28.3 |
|  | Low R/FR | -0.59 | -0.0657 | <0.01 | -6.705 | -26.7 |
| Root length | High R/FR | -0.47 | -0.0235 | <0.01 | -2.395 | -11.4 |
|  | Low R/FR | -0.42 | -0.0204 | 0.06 | -2.078 | -9.8 |
| SPAD | High R/FR | 0.57 | 0.0294 | <0.01 | 3.001 | +9.1 |
|  | Low R/FR | 0.69 | 0.0370 | <0.01 | 3.774 | +11.3 |
| Dry biomass | High R/FR | 0.19 | 2.09e-04 | 0.59 | 0.021 | +8.0 |
|  | Low R/FR | 0.07 | 7.06e-05 | 0.85 | 0.007 | +2.6 |
| Height/stem diameter | High R/FR | 0.57 | 0.0538 | <0.01 | 5.483 | +11.1 |
|  | Low R/FR | 0.62 | 0.0599 | <0.01 | 6.108 | +9.9 |
| Root length/height | High R/FR | -0.61 | -0.0015 | <0.01 | -0.151 | -17.5 |
|  | Low R/FR | -0.54 | -9.59e-04 | <0.01 | -0.098 | -13.3 |
